# Supplementary material for: Establishing the contribution of active histone methylation marks to the aging transcriptional landscape of Drosophila photoreceptors
Source: Sci Rep. 2023 Mar 29;13:5105. doi: 10.1038/s41598-023-32273-5 (PMC10060402; doi:10.1038/s41598-023-32273-5)

SUPPLEMENTAL DATA:

Supplemental Figure 1. A. Experimental diagram of chromatin immunoprecipitation coupled with incorporation of exogenous *Arabidopsis* chromatin for spike-in normalization. **B.** PCA of spike-in and H3-normalized H3K4me3 and H3K36me3 samples during aging. **C.** Gene metaplots showing individual replicates used to compute the average gene metaplot in Figure 1D. **D.** Gene metaplots showing spike-in and H3-normalized H3K4me3 and H3K36me3 samples during aging.

Supplemental Figure 2. Boxplots of expression values (TPM, transcripts per million relative to day 10, set at one) for H3K4me3 and H3K36me3 writers and erasers. Differential expression analysis was used to determine statistically significant changes in gene expression between D10 and D40 samples. ns., not significant, * adj.p-val<0.05, ** adj.p-val<0.005.

Supplemental Figure 3. A. Gene metaplot showing ATAC-seq signal (CPM) over gene bodies in photoreceptors expressing shSet2 versus shControl at day 10. **B.** MA plot showing significant changes in chromatin accessibility determined by ATAC-seq at peaks in photoreceptors expressing shSet2 versus shControl at day 10.

Supplemental Figure 4. Full panels for western blot showing H3K36me3 and H3 levels in larvae expressing a shRNA against Set2 (shSet2) or mCherry (shCtrl) under tubGal4 control as shown in Figure 3B.

Supplemental Figure 5. A. Genomic overlap analysis of spliced exons identified using rMATS. Only statistically significant exons (FDR<5%) were selected for overlap analysis using ChIPseeker. **B.** qPCR analysis of alternatively-spliced exons in control and Set2 knockdown flies. cDNA was prepared from four biological replicates of 20 eyes from D10 male flies. Data is plotted as the ratio of the level of transcript with the alternatively-spliced exon included to level of the transcript without the exon included. ns., not significant, * p-val<0.05, Student's t-test.

Supplemental Table 1: Differentially expressed genes in shSet2 photoreceptors versus shControl.

Supplemental Table 2: Differential splicing events in shSet2 photoreceptors versus shControl.

Supplemental Table 3: Differential splicing events in day 10 versus day 40 photoreceptors.

Supplemental Table 4: qPCR primers used in this study.

Jauregui-Lozano_suppFig1

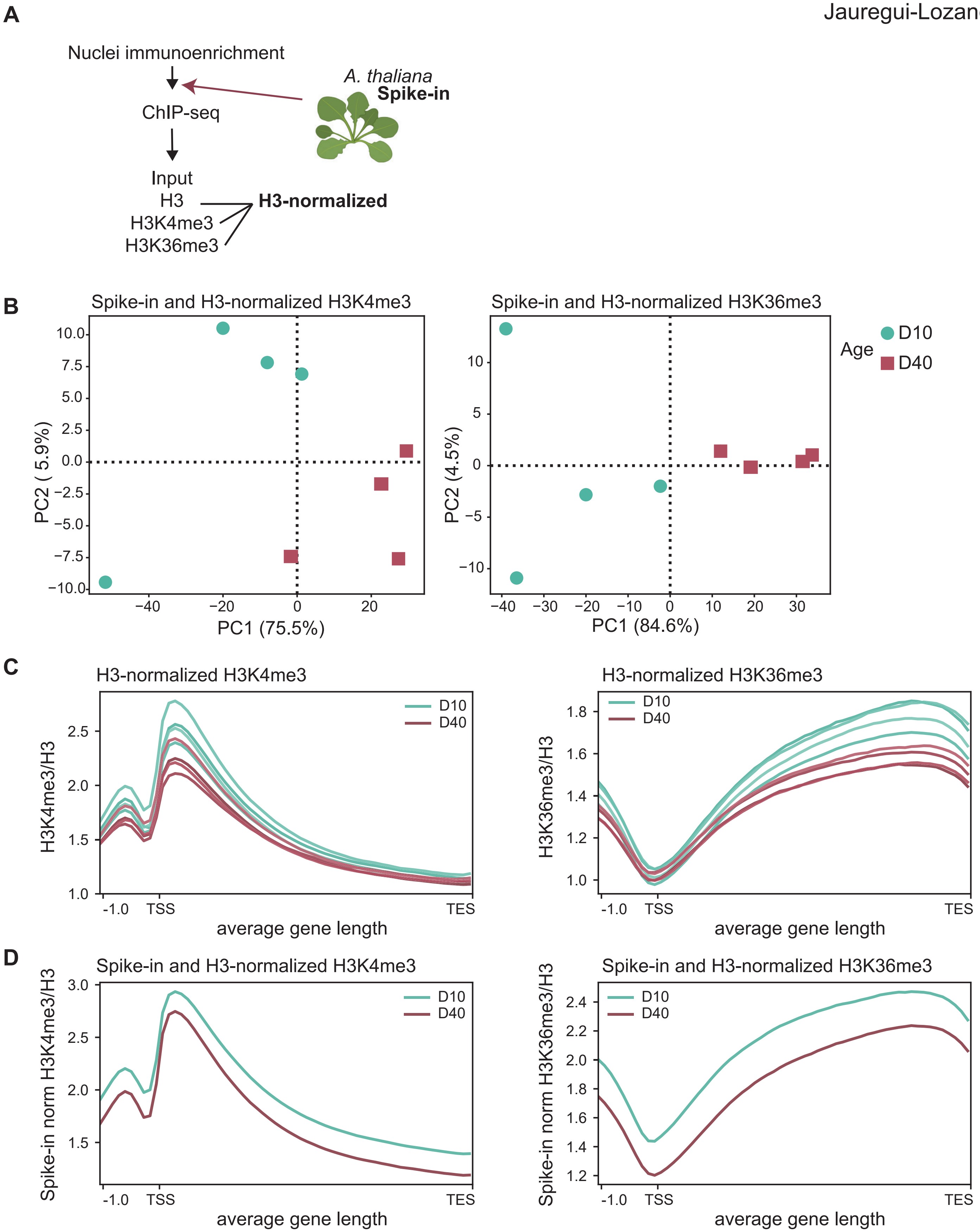

Jauregui-Lozano_suppFig2

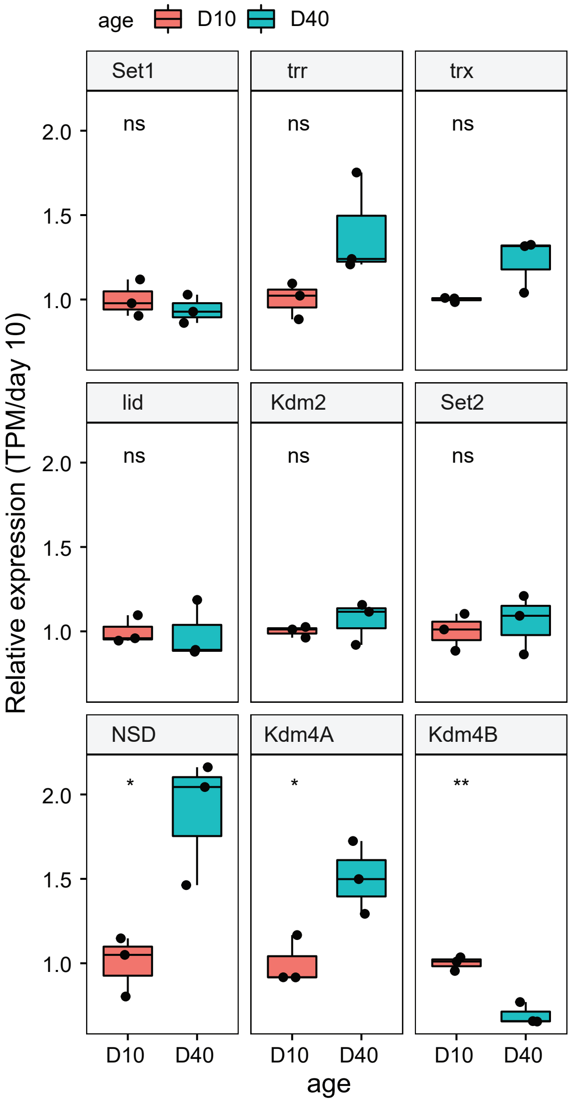

A

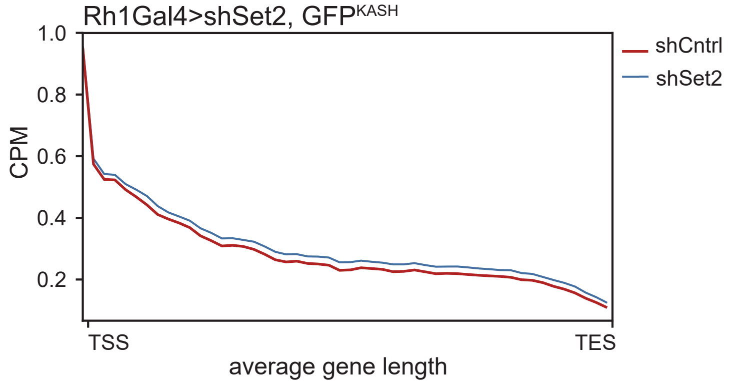
Rh1Gal4>shSet2, GFP^KASH
1.0
0.8
0.6
0.4
0.2
CPM
shCntrl
shSet2
TSS
TES
average gene length

B

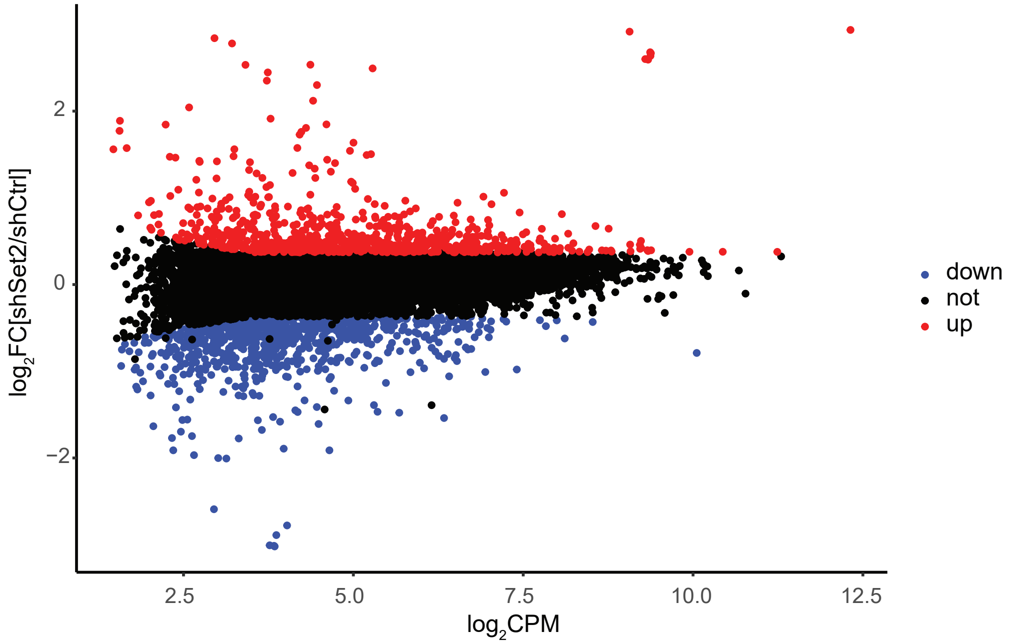
2
0
−2
log2FC[shSet2/shCtrl]
2.5
5.0
7.5
10.0
12.5
log2CPM
down
not
up

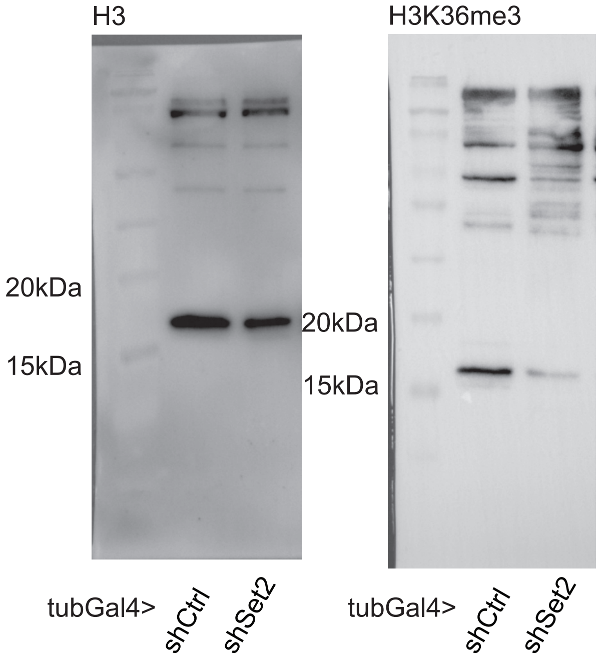
H3
20kDa
15kDa
tubGal4>
shCtrl
shSet2
H3K36me3
20kDa
15kDa
tubGal4>
shCtrl
shSet2

Jauregui-Lozano_suppFig5

A

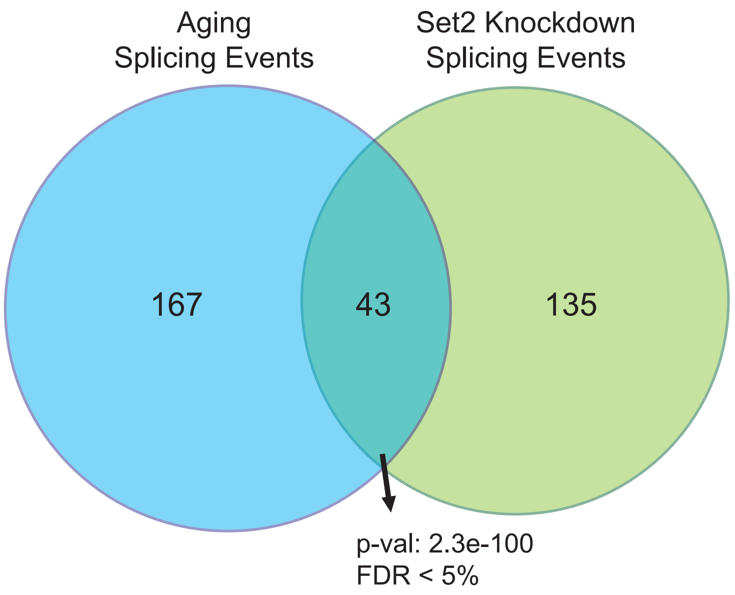

B

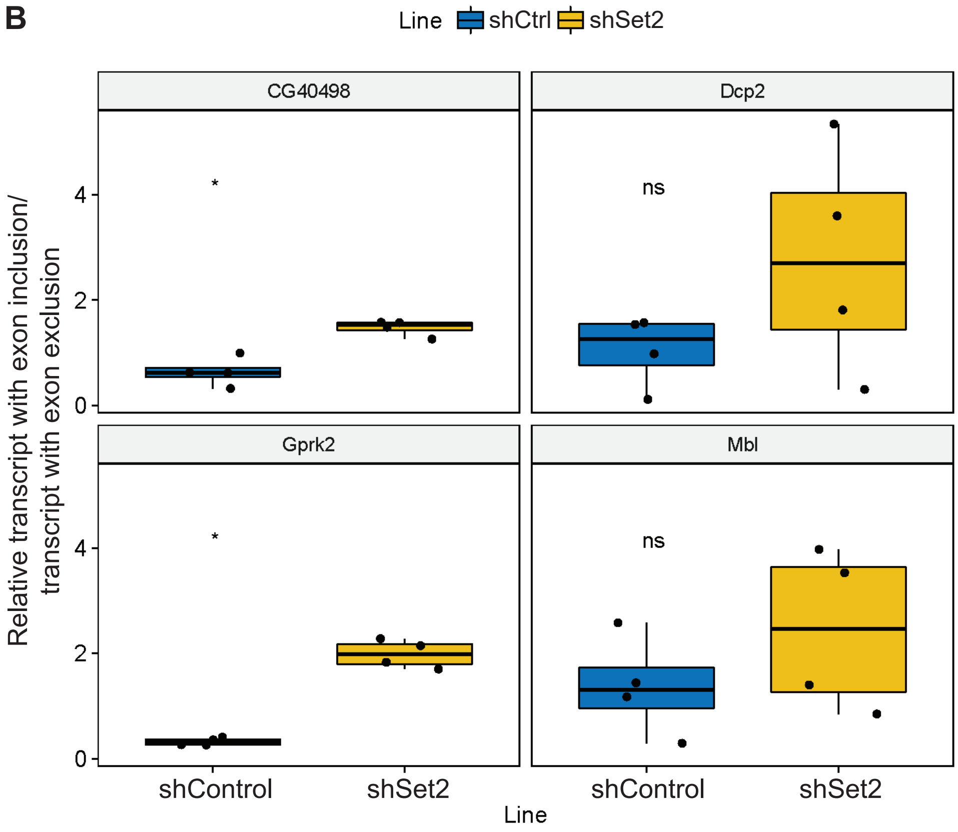

Supplement: Supplementary file 1 — Supplementary Information. [file 41598_2023_32273_MOESM1_ESM.pdf]
